# Weighted Gene Co-Expression Network Coupled with a Critical-Time-Point Analysis during Pathogenesis for Predicting the Molecular Mechanism Underlying Blast Resistance in Rice

**DOI:** 10.1186/s12284-020-00439-8

**Published:** 2020-12-11

**Authors:** Dagang Tian, Zaijie Chen, Yan Lin, Ziqiang Chen, Khuynh The Bui, Zonghua Wang, Feng Wang

**Affiliations:** 1 Biotechnology Research Institute, Fujian Provincial Key Laboratory of Genetic Engineering for Agriculture, Fujian Academy of Agricultural Sciences, Fuzhou, 350003 China; 2 State Key Laboratory of Ecological Pest Control for Fujian and Taiwan Crops, College of Life Science, Fujian Agriculture and Forestry University, Fuzhou, China; 3 Graduate School of Bioagricultural Sciences, Nagoya University, Nagoya, Japan

**Keywords:** Rice, Blast, RNA-seq, *Piz-t*, Clustering, PCA, WGCNA

## Abstract

**Background:**

Rice blast, caused by the ascomycete fungus *M. oryzae*, is one of the most important diseases of rice. Although many blast resistance (*R*) genes have been identified and deployed in rice varieties, the molecular mechanisms responsible for the *R* gene-mediated defense responses are yet not fully understood.

**Results:**

In this study, we used comparative transcriptomic analysis to explore the molecular mechanism involved in Piz-t-mediated resistance in a transgenic line containing *Piz-t* (NPB-Piz-t) compared to Nipponbare (NPB). Clustering and principal component analysis (PCA) revealed that the time-point at 24-h post inoculation (hpi) was the most important factor distinguishing the four time-points, which consisted of four genes of mitogen-activated protein kinases (MAPKs) signaling pathway, one gene related to WRKY DNA-binding domain containing protein, five pathogenesis-related protein (OsPR1s) genes, and three genes of *R* proteins involving in the most significant protein-protein interaction (PPI) pathway. Using weighted gene co-expression network analysis (WGCNA) to investigate RNA-seq data across 0, 24, 48, and 72 hpi, nine modules with similar patterns expression pattern (SEP) and three modules with differential expression pattern (DEP) between NPB-Piz-t and NPB across 0, 24, 48, and 72 hpi with KJ201 (referred to as Piz-t-KJ201 and NPB-KJ201) were identified. Among these the most representative SEP green-yellow module is associated with photosynthesis, and DEP pink module comprised of two specific expressed nucleotide-binding domain and leucine-rich repeat (*NLR*) genes of LOC_Os06g17900 and LOC_Os06g17920 of *Pi2/9* homologous, three *NLR* genes of LOC_Os11g11810, LOC_Os11g11770, and LOC_Os11g11920 which are putatively associated with important agronomic traits, and a B3 DNA binding domain containing protein related genes (LOC_Os10g39190). Knockout of LOC_Os10g39190 via CRISPR-Cas9 resulted in plant death at the seedling stage.

**Conclusions:**

The research suggested that *Piz-t* and multiple *NLR* network might play important roles in the regulation of the resistance response in the Piz-t-KJ201 interaction system. The identified genes provide an *NLR* repository to study the rice-*M. oryzae* interaction system and facilitate the breeding of blast-resistant cultivars in the future.

## Background

Rice blast is a major disease threatening global rice production, causing a 10–30% global crop yield losses. Utilization of rice-blast resistant cultivar is the most economical and environment-friendly way of minimizing crop losses. Currently, over 100 rice-blast R genes have been mapped and at least 32 have been characterized and cloned (Xiao et al. 2020; Zhao et al. 2018). However, only few broad-spectrum and durable resistant cultivars have been bred due to the highly complex and dynamic process associated with blast resistance response (Ashkani et al. 2016).

The completion of the whole genome sequences for rice and *M. oryzae* has made the pathosystem become a premier model for studying plant-fungal interactions, which increases our understanding of the mechanisms underlying fungal infections. The major members involved in rice-*M. oryzae* interactions include pattern-recognition receptors (PRRs) and *NLR* genes from rice, which trigger a diverse array of immune responses including energy metabolism, pathogen recognition, defense related proteins, hormone signaling, ROS, and redox homeostasis, especially on the changes of genes expression and transcriptional reprogramming. Of these, *R* genes are well studied as the famous gene for gene resistance; meanwhile, a large NLR proteins immune signaling network responses to invading pathogens and confers more durable resistance than single race-specific *R* gene (Wu et al. 2017). Understanding of these genes mediated defense response to blast can be useful in helping resistance breeding.

Previously, proteome and transcriptome analyses have revealed many genes involving in the defense responses of rice to *M. oryzae* (Wei et al. 2013; Wang et al. 2014; Zhang et al. 2016; Jain et al. 2017; Tian et al. 2018), however, little is known about the potential connection among these genes. Recently, several approaches have been developed to look at genes with similar expression patterns which may participate in specific biological functions. One approach is the method named WGCNA, which was implemented as a R package to identify significant associated genes modules with similar expressions. This reduces the dimension of complex data and simplifies it into several modules, thereby providing an overview of gene-gene inter-relationships at the system level. In addition, clustering is a useful exploratory technique for analysis of gene expression data, and PCA can help to reduce the dimensionality of the data set by transforming to a new set of variables to summarize the features of the data. Thus, integrating these two analysis techniques to analyze the four time-point data can contribute to determine the critical one (Yeung and Ruzzo 2001).

The rice blast *R* gene *Piz-t* is a member of the *Pi2/9* multi-allelic gene family on chromosome 6 (Qu et al. 2006), which follows a gene-for-gene fashion to the *M. oryzae AVR* gene *AvrPiz-t* (Li et al. 2009a, b). This gene was once widely used in breeding programs for increasing resistance to *M. oryzae* (Zhou et al. 2006; Tian et al. 2020). Numerous studies also have examined the interaction profiles of Piz-t-AvrPiz-t in rice (Park et al. 2012, 2016; Wang et al. 2016; Tang et al. 2017; Tian et al. 2018; Bai et al. 2019; Zhang et al. 2020). However, transcriptome profiling underlying the Piz-t-mediated rice resistance to blast fungus remains largely unknown.

Generally, the whole infection process begins at the first time infected at 24 hpi, and then invades the neighboring cells at 48 hpi (Cao et al. 2016). Subsequently, rice blast disease lesions will emerge about 72 hpi (Wilson and Talbot 2009). The objective of this study then is to identify a set of candidate defense genes mediated by *Piz-t* with *M. oryzae* using the clustering, PCA, and WGCNA approach across the four time-points. Notably, a larger number of *R* genes and regulators were identified during pathogenesis. The identified genes can be further studied to reveal the molecular mechanisms underlying rice-blast interactions in the future.

## Results

### RNA-Seq and Validation of RNA-Seq Data by qRT-PCR

**Fig. 1:**
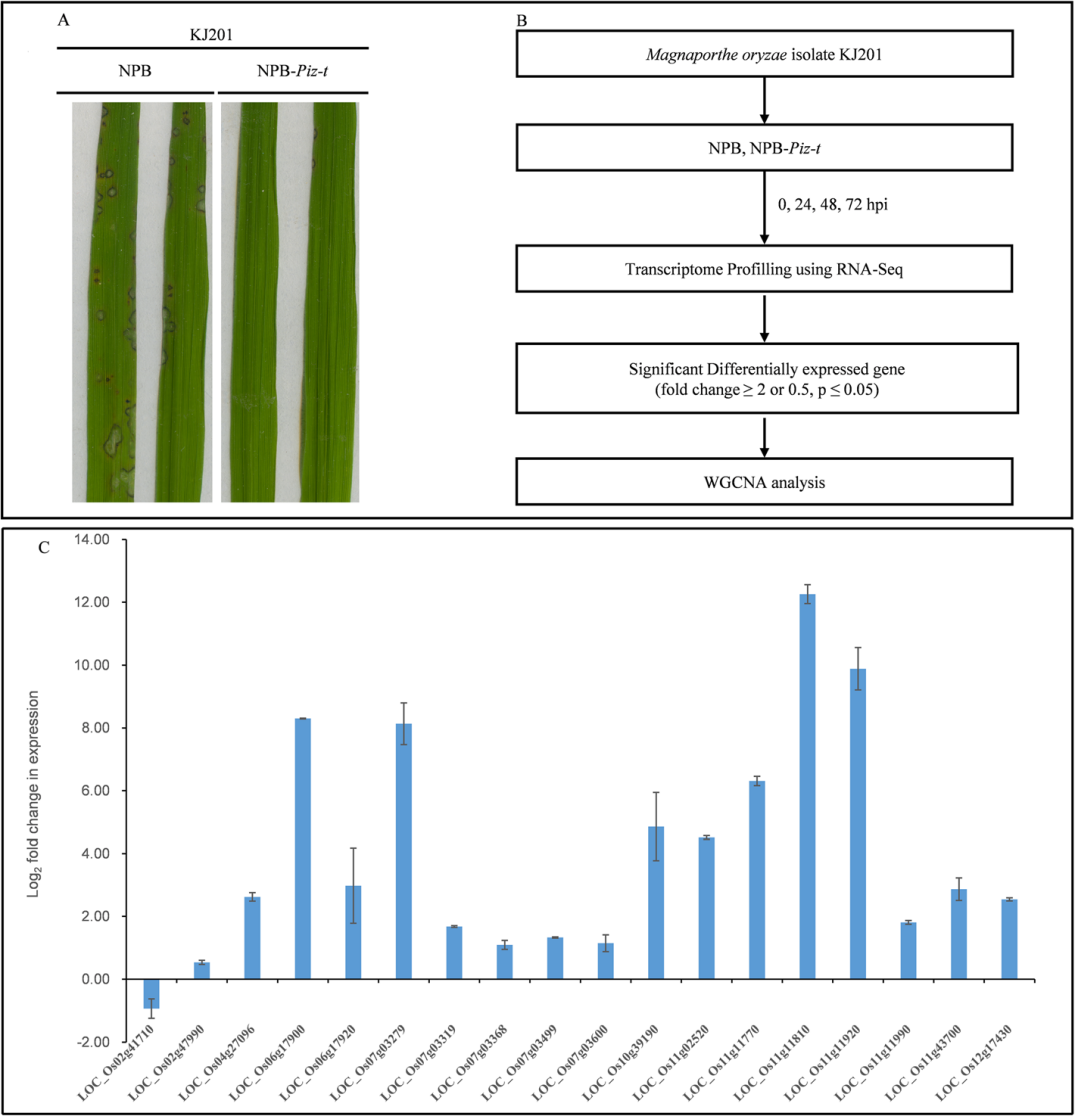
Disease phenotype of NPB and NPB-Piz-t inoculated with KJ201 and the workflow of the transcriptomic experiment and validation for WGCNA. **a** Phenotype of NPB and NPB-Piz-t inoculated with KJ201. **b** The workflow for leaf transcriptome of Piz-t-KJ201 and NPB-KJ201 using RNA-Seq. **c** The qRT-PCR validation of the differentially expressed gene between NPB-Piz-t and NPB inoculated with KJ201 in the PPI pathway at 24 hpi. The data were log2 transformed for FC

Leaf tissues were collected from each rice line at 0, 24, 48, and 72 hpi for RNA-seq. Some plants were retained for investigation of disease progression. There were some spindle-shaped lesions on NPB leaves and no obvious disease phenotype on Piz-t-KJ201 leaves after 7 days of incubation according to a disease rating of 5 (on a 0–5 scale) (Mackill and Bonman 1992) (Fig. 1a). RNA-Seq analysis of Piz-t-KJ201 and NPB-KJ201 were conducted as shown in Fig. 1b. A total of 1,242,379,052 high-quality reads were generated, and over 90% of the reads in each sample successfully aligned to RGAP7, with unique mapped reads at > 85%, and multiple mapped reads or fragments were < 6%. To confirm the gene expression patterns identified from the RNA-seq data, the transcript levels of 18 important genes were examined and validated by qRT-PCR (Additional Table 1). All the gene expression patterns obtained were found in consistency with the RNA-seq data (Fig. 1c).

**Fig. 2:**
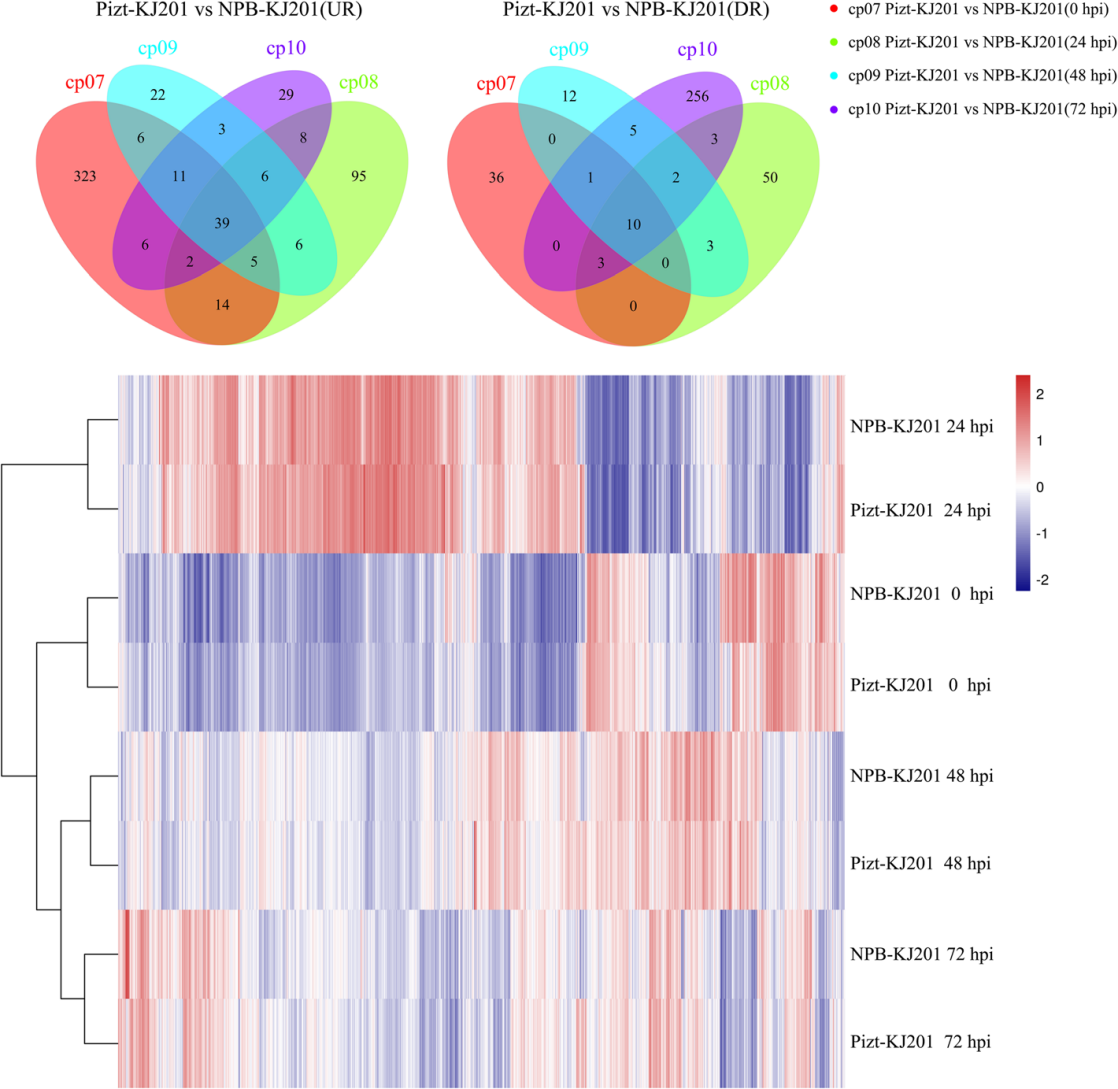
Expression profile of resistant (Piz-t-KJ201) and susceptible (NPB-KJ201) lines at 0, 24, 48, and 72 hpi. **a** Venn diagram showing the up-regulated (UR) DEGs of Piz-t-KJ201 compared to NPB-KJ201 at 0, 24, 48, and 72 hpi. **b** Venn diagram showing the down-regulated (DR) DEGs of Piz-t-KJ201 compared to NPB-KJ201 at 0, 24, 48, and 72 hpi. **c** Clustering significant (FDR adjusted *p* ≤ 0.05 & log2 fold change ≥2) differentially expressed loci of Piz-t-KJ201 and their respective log2 fold change in NPB-KJ201. Red represents higher expression loci and blue represents lower expression loci

**Fig. 3:**
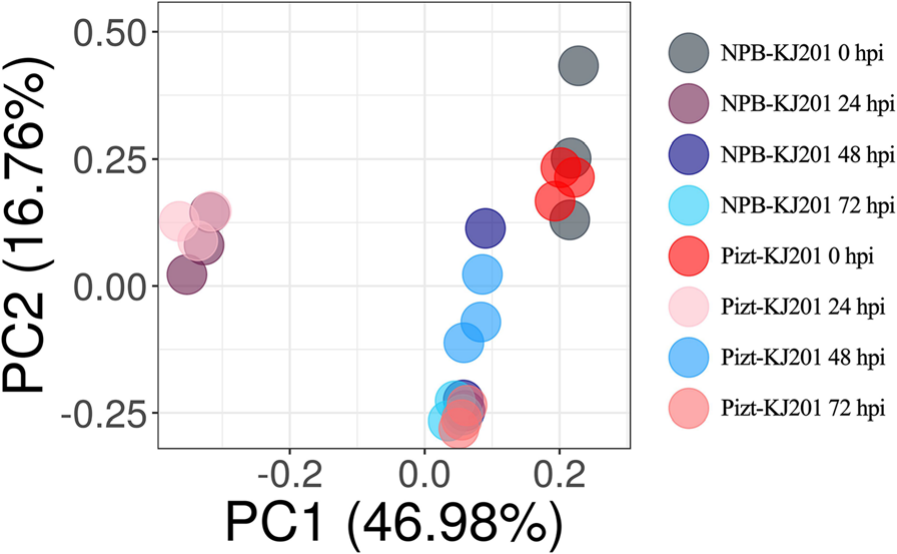
PCA analysis of DEGs in Piz-t-KJ201 and NPB-KJ201 at 0, 24, 48, and 72 hpi with KJ201

Results from the venn diagram showed that the up-regulated differentially expressed genes (DEGs) were 406, 175, 98, and 104, and the down-regulated ones with 50, 71, 33, and 280 at 0, 24, 48, and 72 hpi, respectively, and 49 common DEGs at the four time-points (Fig. 2a,b). Clustering analysis also revealed that those DEGs at the four time-points have consistent expression levels among their three replicates, respectively. Although part of those genes expression levels at Piz-t-0hpi.3 were different with that at Piz-t-0hpi.1 and Piz-t-0hpi.2, they had higher level of consistency with Piz-t-0 hpi compared with NPB-0 hpi (Additional Fig. 1.A, B, C, D). Further comparisons of the DEGs across the four time-points showed that most of the DEGs were up-regulated at 0 hpi and down-regulated at 24 hpi. In contrast, almost opposite expression patterns were seen between 0 and 24 hpi. Also, the DEGs at 24 hpi were significantly different from the other three time-points as shown by Fig. 2c. PCA on 24 group data related to Piz-t-KJ201 and NPB-KJ201 with three replicates revealed that the first principal component (PC), PC1, provided the most information on time-points that determine expression difference (Fig. 3), suggesting that the time-point at 24 hpi is the most significant factor that triggers global defense response in gene expression.

Previous studies suggested that the pathogens finish the first time infected at 24 hpi, accordingly, the rice activates defense responses against *M. oryzae* before 24 h (Bagnaresi et al. 2012; Jain et al. 2017). Thus, this time-point at 24 hpi is the most intriguing for further extensive exploration.

### Differentially Transcriptional Profiles between Piz-t-KJ201 and NPB-KJ201 at 24 hpi

At 24 hpi, 175 and 71 genes were identified to be up-regulated and down-regulated in Piz-t-KJ201 compared to NPB-Piz-t, respectively (Supplemental Table 2). To provide valuable insight into the functional associations of the 246 DEGs at the critical time-point, GO analysis of these DEGs showed that the most significant categories of biological processes were associated with auxin catabolic processes, oxazole or thiazole biosynthetic and metabolic processes. The most significant cellular component categories correlated with extracellular region, intrinsic and integral component of plasma membrane, and plasma membrane, and the most molecular function categories related to ADP binding, monooxygenase activity, oxidoreductase activity, and ion binding (Additional Fig. 2A). Kyoto Encyclopedia of Genes and Genomes (KEGG) analysis revealed that the DEGs functions were mainly involved in PPI, lysine degradation, MAPK signaling pathway, cAMP signaling pathway (Additional Fig. 2B). Several transcription factors (TF) such as FAR1, WRKY, NAC, and bHLH were also significantly enriched in the critical time-point (Table 1).

**Table 1:** The differentially expressed genes of transcription factors (TFs) and PPI pathway at 24 h post inoculation

Group	Gene_name	Description	Comparison (Piz-t-KJ201 vs NPB-KJ201)	Categorize by function
TF	LOC_Os01g06640	Os01g0159800, basic helix-loop-helix, putative, expressed	5.01/2.17	Dof
	LOC_Os01g11910	Os01g0218100, basic helix-loop-helix, putative, expressed	14.72/6.42	WRKY
	LOC_Os02g14490	Os02g0241200, MYB family transcription factor, putative, expressed	0.27/1.04	B3
	LOC_Os02g15350	Os02g0252400, dof zinc finger domain containing protein, putative, expressed	6.06/0	MYB
	LOC_Os02g36880	Os02g0579000, no apical meristem protein, putative, expressed	4.88/1.88	ERF
	LOC_Os05g03740	Os05g0128000, transcription factor TF2, putative, expressed	1.72/0.73	FAR1
	LOC_Os05g50610	Os05g0583000, WRKY8, expressed	3.91/0.95	WRKY
	LOC_Os08g14880	Os08g0246800, transposon protein, putative, unclassified, expressed	21.81/9.97	NAC
	LOC_Os09g28440	Os09g0457900, AP2 domain containing protein, expressed	0.81/0	NAC
	LOC_Os10g34884	Os10g0490300, RIPER7 - Ripening-related family protein precursor, expressed	0/0.03	bHLH
	LOC_Os10g39190	Os10g0537100, B3 DNA binding domain containing protein, expressed	10.11/0.63	G2-like
	LOC_Os11g02520	Os11g0117400, WRKY104, expressed	3.05/0.25	FAR1
	LOC_Os11g35390	Os11g0558200, MYB family transcription factor, putative, expressed	1.78/4.70	Trihelix
	LOC_Os12g03050	Os12g0123800, no apical meristem protein, putative, expressed	0.57/0.08	bHLH
PPI	LOC_Os02g41710	Os02g0627700, cyclic nucleotide-gated ion channel, putative, expressed	0.09/0.47	MAPK signaling pathway
	LOC_Os02g47990	AP014958, retrotransposon protein, putative, unclassified, expressed	0.14/0.03	MAPK signaling pathway
	LOC_Os04g27096	Os04g0340100, expressed protein	3.53/1.00	MAPK signaling pathway
	LOC_Os06g17900	Os06g0286700, disease resistance protein RPM1, putative, expressed	35.65/0.50	MAPK signaling pathway
	LOC_Os06g17920	Os06g0287000, NBS-LRR disease resistance protein, putative, expressed	10.47/0.03	WRKY DNA-binding domain containing protein, expressed
	LOC_Os07g03279	Os07g0124900, SCP-like extracellular protein, expressed	51.58/15.30	OsPR1#071
	LOC_Os07g03319	Os07g0125201, SCP-like extracellular protein, expressed	2.49/0.56	OsPR1#072
	LOC_Os07g03368	Os07g0124900, SCP-like extracellular protein, expressed	113.56/34.77	OsPR1#072
	LOC_Os07g03499	Os07g0125201, SCP-like extracellular protein, expressed	3.47/0.74	OsPR1#072
	LOC_Os07g03600	Os07g0127700, SCP-like extracellular protein, expressed	1.39/0.28	OsPR1#074; PR-1a
	LOC_Os07g12240	AP014963, EF hand family protein, putative, expressed	0.13/0	R protein, expressed
	LOC_Os11g02520	Os11g0117400, WRKY104, expressed	3.05/0.25	R protein, expressed
	LOC_Os11g11770	Os11g0224900, disease resistance protein RPM1, putative, expressed	8.44/0.25	R protein, expressed
	LOC_Os11g11810	Os11g0225300, NBS-LRR disease resistance protein, putative, expressed	5.60/0	R protein, expressed
	LOC_Os11g11920	Os11g0226400, resistance protein, putative, expressed	7.17/0	R protein, expressed
	LOC_Os11g11990	Os11g0227100, NB-ARC domain containing protein, expressed	5.78/2.51	R protein, expressed
	LOC_Os11g43700	Os11g0657900, RGH1A, putative, expressed	1.23/0.54	R protein, expressed
	LOC_Os12g17430	AP014968, NBS-LRR disease resistance protein, putative, expressed	4.01/0.54	R protein, expressed

Next, we selected the genes of the significant PPI pathway for further analysis. Notably, four genes of MAPK signaling pathway, one WRKY-related gene, five PR1 genes, and genes of eight R proteins were involved in this pathway. All of these genes had significantly expressed at higher levels in Piz-t-KJ201 compared to NPB-KJ201 except LOC_Os02g41710, which had higher expression level in NPB-KJ201 (Table 1, Fig. 1c). Interestingly enough, the expression ratios of LOC_Os06g17920 and LOC_Os06g17900 between the Piz-t-KJ201 and NPB-KJ201 were very large, and both of these two genes had small amount of expression levels in the NPB-KJ201 across the four time-points (Table 2, Fig. 1c). In a similar vein, the other four genes also had higher or specific expression in Piz-t-KJ201 (Table 1, Fig. 1c).

**Table 2:** The specific differentially expressed genes in the pink module

Gene_name	Description	Comparison (Piz-t-KJ201/NPB-KJ201)			
		0 hpi	24 hpi	48hpi	72 hpi
LOC_Os06g17900	Os06g0286700, disease resistance protein, putative, expressed	38.87/0.78	35.65/0.50	35.94/0.55	28.08/0.57
LOC_Os06g17920	Os06g0287000, NBS-LRR disease resistance protein, putative, expressed	11.66/0.16	10.47/0.03	11.19/0	7.93/0.07
LOC_Os10g39190	Os10g0537100, B3 DNA binding domain containing protein, expressed	3.76/0.11	10.11/0.63	5.08/0.53	7.19/1.15
LOC_Os11g11770	Os11g0224900, disease resistance protein RPM1, putative, expressed	1.44/0.19	8.44/0.25	2.95/0.16	1.85/0.34
LOC_Os11g11810	Os11g0225300, NBS-LRR disease resistance protein, putative, expressed	2.61/0.14	5.60/0	3.10/0.008	3.77/0.08
LOC_Os11g11920	Os11g0226400, resistance protein, putative, expressed	3.66/0.25	7.17/0	4.66/0.02	6.30/0.22

As the MAPKs are conserved signaling molecules that transduce pathogen stimuli into intra-cellular responses, those up-regulated MAPKs in Piz-t-KJ201 contributed to the activation of PR1s and R genes, leading to the synthesis of antimicrobial compounds such as secondary metabolites that prevent pathogen development. Additionally, several active TFs from FAR1, WRKY, NAC, and bHLH families have been demonstrated to be involved in responses to biotic and abiotic stresses (Ramamoorthy et al. 2008), which were implicated in the regulation of transcriptional reprogramming associated with early response to *M. oryzae*. Taken these genes together showed that expanded transcriptional activation may be controlled by *Piz-t* in the PPI pathways during the early response of rice to *M. oryzae*.

### Gene Co-Expression Networks

**Fig. 4:**
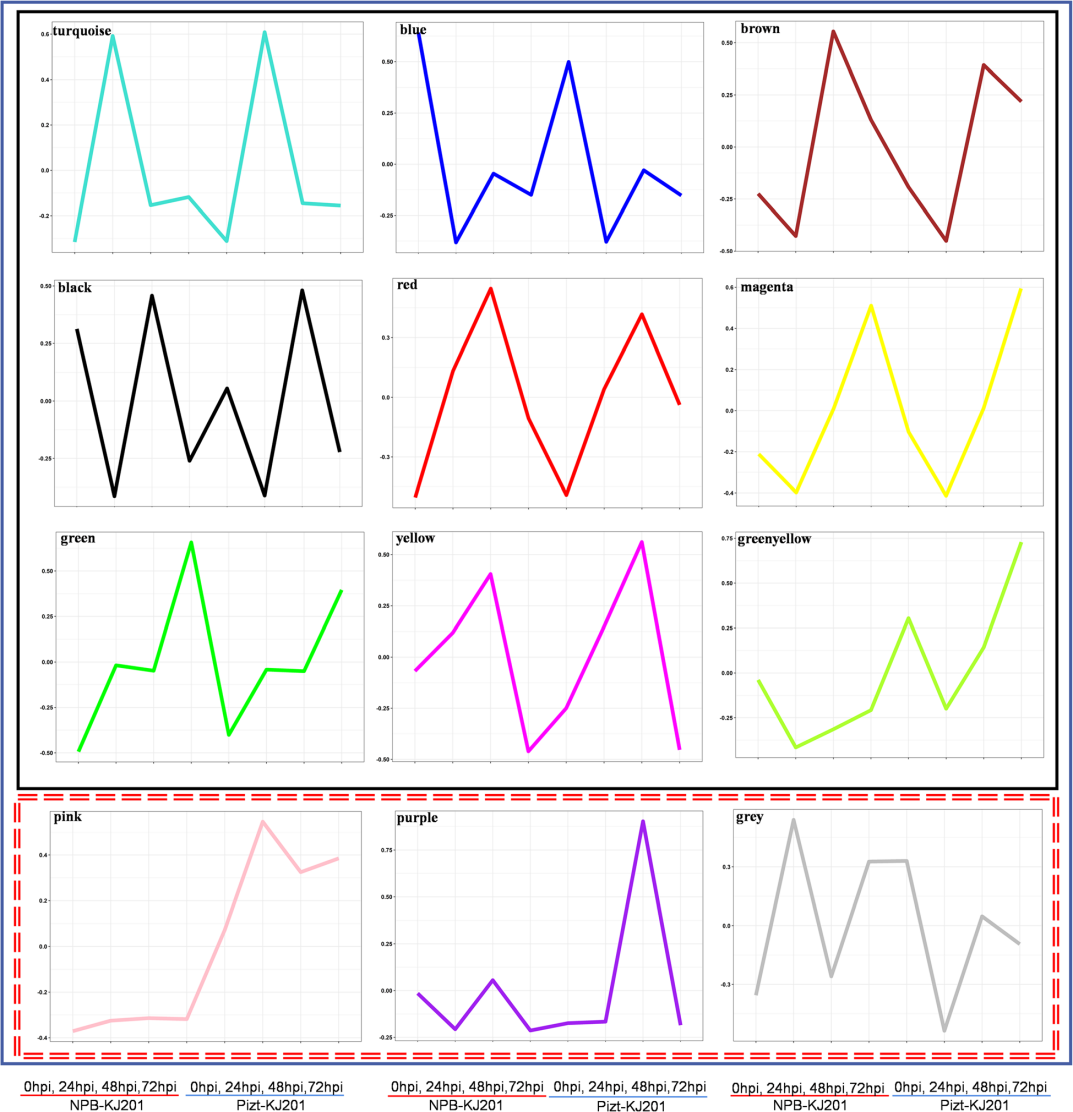
Nine similar (solid circle) and three differentially (double dash line circle) expression patterns module between Piz-t-KJ201 and NPB-KJ201 across 0, 24, 48, and 72 hpi using WGCNA. X-axis signifies NPB-KJ201_0 hpi, NPB-KJ201_24 hpi, NPB-KJ201_48 hpi, NPB-KJ201_72 hpi, Piz-t-KJ201_0 hpi, Piz-t-KJ201_24 hpi, Piz-t-KJ201_48 hpi, Piz-t-KJ201_72 hpi. Vertical coordinates are relative expression values

The WGCNA R package was used to construct a co-expression network consisting of the normalized read counts for the 24 samples based on 33,451 genes. The results showed 12 co-expression modules in gene membership, ranged from as low as three (grey) to as much as 5734 (turquoise) genes, as shown in Additional Tables 3, 4, 5, 6, 7, 8, 9, 10, 11, 12, 13 and 14. These involved genes reflect that the rice-*M. oryzae* interaction is quantitative, and they represent the complexity of a cellular transcription network, from which nine SEP and three DEP modules were enriched, respectively, between NPB-KJ201 and Piz-t-KJ201 (Fig. 4). The nine SEP modules consisted of genes with similar expression profiles between NPB-KJ201 and Piz-t-KJ201 at the four time-points, suggesting the basal similarities between their transcriptional profiles; and the three DEP modules contained those genes with unique expression profiles in NPB-KJ201 or Piz-t-KJ201 at the four time-points, meaning that they may directly associate with resistance response.

The nine SEP was involved in four different types of expression pattern: (i) the turquoise and green modules: up-regulated at 0 and 24 hpi, and then down-regulated at 48 hpi, but up-regulated at 72 hpi again (Additional Tables 3 and 4); (ii) the blue, brown, black, and magenta modules: down-regulated at 0 and 24 hpi, and then up-regulated at 48 hpi, but down-regulated at 72 hpi again (Additional Tables 5, 6, 7, 8); (iii) the red and yellow modules: up-regulated at 0, 24, and 48 hpi, but down-regulated at 72 hpi (Additional Tables 9 and 10); (iv) the green-yellow: down-regulated at 0 and 24 hpi, and up-regulated at 48 and 72 hpi, with an obvious higher expression level in Piz-t-KJ201 across the four time-points compared to NPB-KJ201 (Additional Table 11). On the other hand, the DEP modules were composed of the pink, purple and grey ones. All of these three DEP modules obviously had different expression patterns between Piz-t-KJ201 and NPB-KJ201 across the four time-points (Fig. 4, Supplementary Tables 12, 13, 14). Although the purple model included some obvious DEGs between Piz-t-KJ201 and NPB-KJ201, they didn’t occur at the key time-point of 24 hpi (Supplementary Tables 13). Additionally, the grey modules contained as little as 3 complete reverse regulated DEGs between Piz-t-KJ201 and NPB-KJ201, and they also had lower levels expression in Piz-t-KJ201 compared to NPB-KJ201 at 24 hpi (Supplementary Tables 14). Intriguingly, the pink module contains a set of specific higher DEGs in Piz-t-KJ201 compared to NPB-KJ201, especially at 24 hpi, which suggests that this module involved in DEGs was unique to the resistant response (Supplementary Tables 12).

### Differentially Transcriptional Profiles in the Pink and the Green-Yellow Module

We further analyzed the transcriptional profiles of the representative pink and green-yellow modules, and the results showed that 106 and 34 genes that were differentially regulated between Piz-t-KJ201 and NPB-KJ201 (Additional Tables 11 and 12). Gene ontology (GO) analysis of DEGs in the pink module showed that the most significant molecular function categories identified were ADP binding, adenyl ribonucleotide binding, adenyl nucleotide binding, ion binding, and nucleotide binding. Meanwhile, the most significant biological processes were associated with DNA integration, cell communication, and signaling transduction. In the case of DEGs in the green-yellow module, the most significant molecular function categories were chlorophyll binding, electron transporter and transfer activity, and tetrapyrrole binding. The most significant biological process categories were photosynthetic electron transport in photosystem II, protein-chromophore linkage, and photosynthesis process, while cellular component categories identified include those associated with photosystem, thylakoid, and membrane protein complex. KEGG analysis also revealed that DEGs specific to the pink module were mainly involved in pathways related to PPI, while DEGs specific to the green-yellow module were mainly associated with the photosynthesis (Table 3).

**Table 3:** The number of significant differentially expressed genes in pink and yellow-green present in different GO and KEGG terms

Module	GO Accessions	Term types	Go Terms	Number of DEGs	***P***-value
Pink	GO:0015074	Biological process (BP)	DNA integration	6	8.70E-06
	GO:0007154	Biological process (BP)	cell communication	10	0.00062
	GO:0007165	Biological process (BP)	signal transduction	9	0.00095
	GO:0023052	Biological process (BP)	signaling	9	0.00095
	GO:0043531	Molecular function (MF)	ADP binding	15	2.2E-12
	GO:0032559	Molecular function (MF)	adenyl ribonucleotide binding	23	0.000034
	GO:0030554	Molecular function (MF)	adenyl nucleotide binding	23	0.000035
	GO:0043167	Molecular function (MF)	ion binding	35	0.000056
	GO:0000166	Molecular function (MF)	nucleotide binding	25	0.000057
	ko04626	Pathway	Plant-pathogen interaction	8	0.000883
green-yellow	GO:0009772	Biological process (BP)	photosynthetic electron transport in photosystem II	7	2.70E-17
	GO:0018298	Biological process (BP)	protein-chromophore linkage	8	7.70E-17
	GO:0009767	Biological process (BP)	photosynthetic electron transport chain	8	6.9E-16
	GO:0019684	Biological process (BP)	photosynthesis, light reaction	9	9.20E-15
	GO:0015979	Biological process (BP)	photosynthesis	10	1.00E-13
	GO:0016168	Molecular function (MF)	chlorophyll binding	8	1.6E-16
	GO:0045156	Molecular function (MF)	electron transporter	7	1.30E-15
	GO:0009055	Molecular function (MF)	electron transfer activity	7	9.40E-09
	GO:0046906	Molecular function (MF)	tetrapyrrole binding	8	3.50E-07
	GO:0009521	Cellular component (CC)	photosystem	10	2.00E-16
	GO:0009523	Cellular component (CC)	photosystem II	8	1.40E-13
	GO:0034357	Cellular component (CC)	photosynthetic membrane	10	4.70E-12
	GO:0044436	Cellular component (CC)	thylakoid part	10	7.80E-12
	ko00195	Pathway	Photosynthesis	9	6.53E-11

Five resistance genes of LOC_Os11g11810, LOC_Os06g17920, LOC_Os11g11770, LOC_Os06g17900, and LOC_Os11g11920 involved in PPI pathway and one TF of LOC_Os10g39190 are the most significant element in the pink module (Table 2, Fig. 1c, Additional Fig. 3). Interestingly, LOC_Os06g17900 and LOC_Os06g17920, two putative pseudogenes of the *Pi2/9* homologous showed the first and second highest values, respectively, and three activated NLR genes of LOC_Os11g11810, LOC_Os11g11770, and LOC_Os11g11920 in Piz-t-KJ201 have been reported to associate with important agronomic traits. Another TF of LOC_Os10g39190 perhaps involved in ABA signaling regulation was significantly up-regulated in Piz-t-KJ201 plants across four time-points, especially at 24 hpi (Table 2, Fig. 1c, Additional Fig. 3).

Next, we focused on the functional analysis of LOC_Os10g39190. One gRNA locating in the 345 bp of Os10g053710 was designed to silence this gene (Additional Fig. 4). Although we obtained six CRISPR mutants, but all of these transgenic plants didn’t survive at the seedling stage.

## Discussion

In this study, we showed an approach in evaluating large transcription data using co-expression networks in combination with the critical time-point at 24 hpi analysis. Specifically, we applied this strategy in analyzing gene expression dataset from rice in response to *M. oryzae* infection and identifying genes possibly involved in rice against *M. oryzae,* In this study, we identified several significant DEGs related with TF, MAPK signaling pathway, OsPR1, and R proteins of genes to be possibly involved in the resistance response in the Piz-t-KJ201 interaction, facilitating understanding the interaction of rice-*M. oryzae* and breeding for new resistant cultivars.

### Resistance Response Involved in the Regulation of Photosynthesis

Previously, it has been reported that both pathogen-associated molecular pattern (PAMP) -triggered immunity (PTI) and effector-triggered immunity (ETI) contribute to the resistance response to rice blast (Boller and Felix 2009), and *NLR*-mediated immunity is known to be modulated by many environmental factors such as light (Gao et al. 2020). In the present study, nine SEP between Piz-t-KJ201 and NPB-KJ201 were found, especially green-yellow module, in which the photosystem plays a vital role. Previous studies confirmed that photosystem was easily targeted by different pathogenic species such as bacteria, viruses, fungi and oomycetes to produce cytoplasmic calcium bursts, jasmonic acid, salicylic acid, and reactive oxygen species, resulting in transcriptional reprogramming to mount a full defense response (Fondong et al. 2007; Jelenska et al. 2007; Rodriguez-Herva et al. 2012; Nomura et al. 2012; Li et al. 2014a, b; Petre et al. 2016; Sowden et al. 2018; Liu et al. 2018; Rosas-Diaz et al. 2018). Subsequently, the photosystem needs to be restored in time to prevent or reduce the damage and energy costs, which can be obviously shown in the green-yellow module with higher expression in Piz-t-KJ201.

### PPI Pathway-Related Differentially Expressed Genes at 24 hpi

PPI was the significant affected pathway in both the pink module and the time-point of 24 hpi. Specially for the 24 hpi, LOC_Os02g41710 (CNGC) was the only MAPK signaling pathway gene that had lower expression in Piz-t-KJ201 as compared with NPB-KJ201. This gene negatively regulates flow of extracellular Ca^2 +^ (Monné et al. 2015). Although the other three MAPK signaling pathway genes were up-regulated in Piz-t-KJ201, complete understanding of their roles in rice disease resistance still remains unclear. Interestingly, five OsPR1 encoding SCP-like extracellular protein were identified to be up-regulated in Piz-t-KJ201, suggesting that they may play particular roles in resistance response. Similarly, there were also evidences that the expression of PR1a and PR1b were related to disease resistance (Ponciano et al. 2006).

Several TFs of WRKY, bHLH, AP2/ERF, Dof, MYB, and NAC etc. were significantly differentially expressed between Piz-t-KJ201 and NPB-KJ201. These members of the TF families have been confirmed to be involved in regulating rice defense responses to *M. oryzae* (Licausi et al. 2013; Yokotani et al. 2014; Cheng et al. 2015). Thus, they may play important roles in the regulation of defenseresponsive genes. The WRKY104 was a positive regulator enhancing the resistance to *M. oryzae* by up-regulating pathogenesis- related (PR) genes as also shown in recent reports (Hou et al. 2019). Additionally, the higher expression of *R* genes LOC_Os11g11990, LOC_Os11g43700, and LOC_Os12g17430 in the Piz-t-KJ201 interaction system were also observed, which have also been implicated in stress responses (Zheng et al. 2014).

### Several Putative NLR Pairs with Piz-t Helper to Mediate Immune Signaling

Plant *R* genes detect effector proteins secreted by pathogens by indirectly or directly binding them via effector-targeted host proteins, with a complex scenario of sequential action of “sensor” NLRs to detect pathogen effectors and “helper” NLRs to initiate immune signaling (Wu et al. 2017). The pink module is the most representative DEP, which includes 6 significant DEGs between Piz-t-KJ201 and NPB-KJ201 across the four time-points. The alleles of LOC_Os06g17920 and LOC_Os06g17900 of *Piz-t* locus were regarded as *R* pseudogenes in the rice Nipponbare genome (Wu et al. 2012). However, when the prediction was based on our present study, these two pseudogenes were actually not pseudogenes but always highly expressed in Piz-t-KJ201 compared to NPB-KJ201 (Table 2). Thus, they are maybe functionally redundant with Piz-t as helper NLR activated by sensors NLR. The specific expressions and characteristics of the alleles of LOC_Os06g17920 and LOC_Os06g17900 in Piz-t-KJ201 deserve further explorations.

In addition, LOC_Os11g11810 and LOC_Os11g11770 were identified to be known *Pia* and *RPM1*, respectively (Okuyama et al. 2011; Yoo et al. 2017; Onaga et al. 2017). Previous studies identified that *Pia* encodes a NBS-LRR disease resistance protein that are known to contribute to weak resistance to rice blast (Okuyama et al. 2011; Césari et al. 2013; Jung et al. 2013); while *RPM1* encodes NB-ARC domain containing protein and can be activated by the defense protein RIN4 to sense effector, and then transmit signals downstream (Li et al. 2014a, b), suggesting that the two genes confer to resistance as the sensor NLRs. Moreover, both of LOC_Os11g11810 and LOC_Os11g11770 were identified to be nonsynonymous single nucleotide polymorphism *NLR* genes, and another *NLR* gene of LOC_Os11g11920 was also down-regulated at developmental period in black rice (functional cultivar) (Seol et al. 2016), implying that they likely play an important role in determining agronomic traits differences. This is consistent with the previous report that a complex relationship exist between disease resistance and yield-related components (Wang et al. 2015).

The B3-domain transcription factor abscisic acid-insensitive 3 (ABI3) was a central regulator in ABA signaling (Zhang et al. 2005). However, to date, there are still a few examples of functional validation of the roles being played by B3 in plant defense. Unfortunately, in the functional identification of LOC_Os10g39190 via CRISPR-Cas9, we can’t get the transgenic seed as all of the T_0_ generation of transgenic plants died at the seedling stage. In general, the effect of knocking out 1 member of the paired *NLRs* was more severe in some sensor mutants compared to some helper mutants, which usually led to the death of the mutants (Wang et al. 2019), from which we can infer that LOC_Os10g39190 maybe as a potential important sensor member.

### Validation of the Present Study by Comparing Previous Reports

In our earlier studies, four proteins of a receptor-like protein kinase (gi|59,800,021), a NADP dependent malic enzyme (gi|54,606,800), a putative transaldolase (gi|57,900,129), and a putative bowman birk trypsin inhibitor (gi|53,792,234), three C3HC4-type Ring finger E3 ubiquitin ligase (APIP2, 6, 10), and a bowman-Birk type bran trypsin inhibitor (APIP4) were confirmed to be involved in Piz-t-KJ201 interaction (Park et al. 2012, 2016; Tian et al. 2018; Zhang et al. 2020). In the present study, we observed that a receptor-like protein kinase precursor (LOC_Os10g33040), a NADP-dependent malic enzyme (LOC_Os05g09440), a transaldolase (LOC_Os08g05830), a bowman-Birk type bran trypsin inhibitor precursor (LOC_Os01g03360), and two zinc finger C3HC4 type domain containing protein (LOC_Os01g20910 and LOC_Os12g04590) were also differentially regulated in Piz-t-KJ201 (Additional Table 2). Therefore, parts of the present results were validated by the previous studies. Though we tried to identify the function of LOC_Os10g39190, it’s regretful that the mutant can’t survive at the seedling stage.

This research reveals that many genes are involved in the defense network of Piz-t-KJ201, consistent with previous observations (Park et al. 2012, 2016; Wang et al. 2019; Bai et al. 2019; Zhang et al. 2020). These genes and TFs identified in the present study were of high biological relevance. It was their pairs and interactive *NLR* network that confer blast resistance in the Piz-t-KJ201 interaction as described in the former researches (Wu et al. 2017; Baggs et al. 2017; Wang et al. 2019).

## Conclusion

In this study, we identified that the 24 hpi is the critical time-point during early stage pathogenesis, from which four genes of MAPKs signaling pathway, one gene related to WRKY DNA-binding domain containing protein, five OsPR1s genes, and three genes of R proteins were confirmed involved in the significant PPI pathway. In addition, nine SEP modules and three DEP modules between NPB-Piz-t and NPB were also identified, among which the most representative SEP green-yellow module is associated with photosynthesis, and DEP pink module comprised of two specific expressed *NLR* genes of LOC_Os06g17900 and LOC_Os06g17920 of the *Pi2/9* homologs, three *NLR* genes of LOC_Os11g11810, LOC_Os11g11770, and LOC_Os11g11920 which are putatively associated with important agronomic traits, and a B3 DNA binding domain containing protein related genes (LOC_Os10g39190). Knockout of LOC_Os10g39190 via CRISPR-Cas9 was done, however, transgenic plants failed to survive at the seedling stage. It suggested that *Piz-t* with these *NLRs* and regulators network might play important roles in the regulation of the resistance response in the Piz-t-KJ201 interaction system. The identified genes provide a valuable resource to study the rice-*M. oryzae* interaction system and facilitate breeding of new blast-resistant cultivars in the future.

## Methods

### Materials and Methods

#### Plant Materials and Blast Isolates

Rice (*Oryza sativa* L.) lines, including NPB and its transgenic line with *Piz-t* (NPB-*Piz-t*), and the *M. oryzae* isolates KJ201 was used in this study (Tian et al. 2018). Rice plants were grown in the greenhouse and kept under natural conditions about 2 ~ 3 weeks (3–4 leaves). Spores concentration in the suspension was adjusted to 5 × 10^5^ conidia/mL. After spray-inoculated, the seedlings were maintained in the dark for 24 h at 28 °C and then kept under high humidity over 95% for about a week for symptom evaluation.

Leaf tissues were collected from each rice line at 0, 24, 48, and 72 hpi and frozen in liquid nitrogen. Some leaves of NPB-KJ201 showed obvious disease lesion, while Piz-t-KJ201 had no disease symptoms at 7 days post inoculation (Fig. 1a).

#### Sample Preparation

The total RNA from the infected leaves was extracted using the RNAeasy kit (Qiagen, Germany) and treated with an RNase-Free DNase Set (Qiagen, Germany), according to the manufacturer’s instructions. The RNA quality, library construction and size were assayed using a 2100 Bioanalyzer system (Agilent, USA). The libraries were synthesized using the TruSeq RNA Sample Preparation v2 kit (Illumina, USA). Total RNA from each treatment was pooled and then libraries were constructed as showed in Fig. 1b and used for sequencing. The samples were run in the NovaSeq system and raw sequences of paired 150-bp were obtained.

#### Sequences Processing and Analysis

FastQC was used to estimate the raw reads and assess their quality. Trimming of reads was carried in Trimmomatic and reads containing contaminant primer/adapters and long stretches of poor-quality bases were removed. Each sample was mapped back onto *O. sativa* reference genome from the MSU Rice Genome Annotation Project (MSU Rice Genome Annotation Project Release 7, RGAP7) (Kawahara et al. 2013). Redundancies were removed using Picard suite of tools (http://broadinstitute.github.io/picard/). Alignment was performed using the metrics module of SAMtools (Li et al. 2009a, b). And read counts were estimated using featuresCounts (Anders et al. 2015). Normalization and differential expression analysis was performed using EdgeR (Anders and Huber 2010).

For a given genotype, significant DEGs at each time-point were identified based on fold change (FC) (ratio of number of transcripts in inoculated sample to the number of transcripts in the mock control ≥2 or ≤ 0.5 and a *p*-value < 0.05).

#### Bioinformatics Analysis

Clustering and PCA were done using the R package. Biological processes, cellular components, and molecular functions of DEGs were determined by GO database annotation (http://www.geneontology.org/). Protein signaling pathways were elucidated using the KEGG database (http://www.genome.jp/kegg/ pathway. html). Pathways enriched at *P*-value < 0.05 were considered significant.

#### Co-Expression Network Analysis

Using RSEP version 1.1.1 to quantify expression levels of genes in terms of FPKM (fragments per kilo base of exon per million)1 (Li and Dewey 2011), a common expression matrix for Piz-t-KJ201 and NPB-KJ201 at 0, 24, 48, and 72 hpi was constructed, with three replicates from each condition resulting in a total of 24 samples. Co-expression analysis was performed using R package. The similarity between gene-pairs was computed using a signed Pearson’s correlation matrix, scaled to power β = 11, based on the approximate scale free-topology criterion.

#### Real-Time PCR Validation

Real-time PCR (qRT-PCR) was used to validate DEGs obtained from RNA-Seq. Primers were designed using Primer5 and summarized in Supplementary File 2. The ProtoScript M-MuLV First Strand cDNA Synthesis Kit (NEB) was used to synthesize cDNA. The reaction was performed in the LightCyclerR 480 II PCR system (Roche) with a volume of 10 μl, containing 5 μl of SYBR Green I Master mix, 0.8 μM of forward primer, 0.8 μM reverse primer, 2 μl of 1:5 diluted cDNA template (1–2 μg) and RNAse free water. Actin was used as the internal control. The efficiency of qRT-PCR was calculated in both control and target samples, and FC was calculated using CT method.

## Additional Files

**Additional file 1 **: **Figure S1**. Expression profile of resistant (Piz-t-KJ201) and susceptible (NPB-KJ201) lines with three repeats at 0 hpi (A), 24hpi (B), 48 hpi (C), and 72 hpi (D), respectively.

**Additional file 2 **: **Figure S2**. Bioinformatic analysis of differentially expressed genes in NPB-Piz-t compared to NPB in response to *M. oryzae* KJ201. A, GO analysis of differentially expressed genes of NPB-Piz-t compared to NPB. B, KEGG analysis of differentially expressed genes of NPB-Piz-t compared to NPB. C, Transcript factor of differentially expressed genes. The results are summarized in three main categories: biological process, cellular component, and molecular function.

**Additional file 3 **: **Figure S3**. The qRT-PCR validation of the differentially expressed gene of between NPB-Piz-t and NPB inoculated with KJ201 at 0, 48 and 72 hpi in the pink module. The data were log2 transformed for FC.

**Additional file 4 **: **Figure S4**. Functional analysis of Os10g0537100 using the CRISPR-Cas9 gene editing strategy.

**Additional file 5 **: **Table S1**. Primers used for real-time quantitative PCR used in the study.

**Additional file 6 **: **Table S2**. The information of differentially expressed genes between Piz-t-KJ201 and NPB-KJ201 at 24 hpi.

**Additional file 7 **: **Table S3**. The genes information in turquoise module.

**Additional file 8 **: **Table S4**. The genes information in green module.

**Additional file 9 **: **Table S5**. The genes information in blue module.

**Additional file 10 **: **Table S6**. The genes information in brown module.

**Additional file 11 **: **Table S7**. The genes information in black module.

**Additional file 12 **: **Table S8**. The genes information in magenta module.

**Additional file 13 **: **Table S9**. The genes information in red module.

**Additional file 14 **: **Table S10**. The genes information in yellow module.

**Additional file 15 **: **Table S11**. The genes information in green-yellow module.

**Additional file 16 **: **Table S12**. The genes information in pink module.

**Additional file 17 **: **Table S13**. The genes information in purple module.

**Additional file 18 **: **Table S14**. The genes information in grey module.

## Data Availability

The RNA sequencing data have been submitted to the Sequence Read Archive (SRA) database (https://www.ncbi.nlm.nih.gov/sra) under the accession number PRJNA637691. All data generated or analysed during this study are included in this published article [and its supplementary information files].

## Abbreviations

DEP: Differential patterns of expression
SEP: Similar patterns of expression
WGCNA: Weighted gene co-expression network analysis
PCA: Principal component analysis
NPB: Nipponbare
NPB-Piz-t: Nipponbare transgenic line containing *Piz-t*
NPB-KJ201: Nipponbare inoculated with blast fungus *Magnaporthe oryzae*
Piz-t-KJ201: NPB-Piz-t inoculated with blast fungus *Magnaporthe oryzae*
PPI: Protein-protein interaction
MAPK: Mitogen-activated protein kinase
NLR: Nucleotide-binding domain and leucine-rich repeat-containing
*R*: Resistance
OsPR: Pathogenesis-related protein
DEG: Differentially expression gene
PRR: Pattern-recognition receptor
PAMP: Pathogen-associated molecular pattern
PTI: PAMP-triggered immunity
ETI: Effector-triggered immunity
*Avr*: Avirulence
TF: Transcription factor
GO: Gene ontology
KEGG: Kyoto Encyclopedia of Genes and Genomes
