# Supplementary figures and images for: Weighted Gene Co-Expression Network Coupled with a Critical-Time-Point Analysis during Pathogenesis for Predicting the Molecular Mechanism Underlying Blast Resistance in Rice
Source: Rice (N Y). 2020 Dec 11;13:81. doi: 10.1186/s12284-020-00439-8 (PMC7732884; doi:10.1186/s12284-020-00439-8)

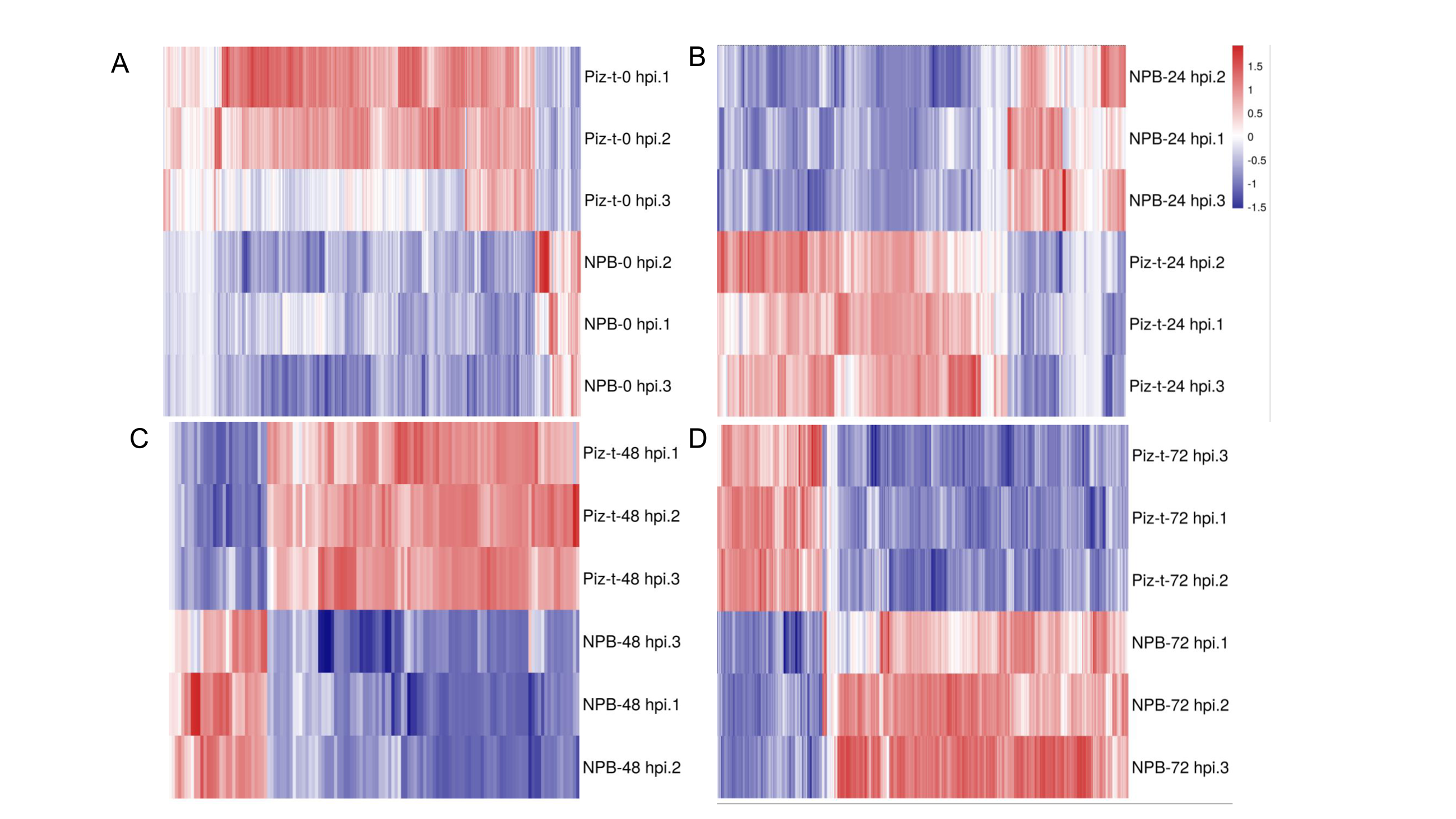

Supplement: Supplementary file 1 — Additional file 1 : Figure S1. Expression profile of resistant (Piz-t-KJ201) and susceptible (NPB-KJ201) lines with three repeats at 0 hpi (A), 24hpi (B), 48 hpi (C), and 72 hpi (D), respectively. [file 12284_2020_439_MOESM1_ESM.tif]

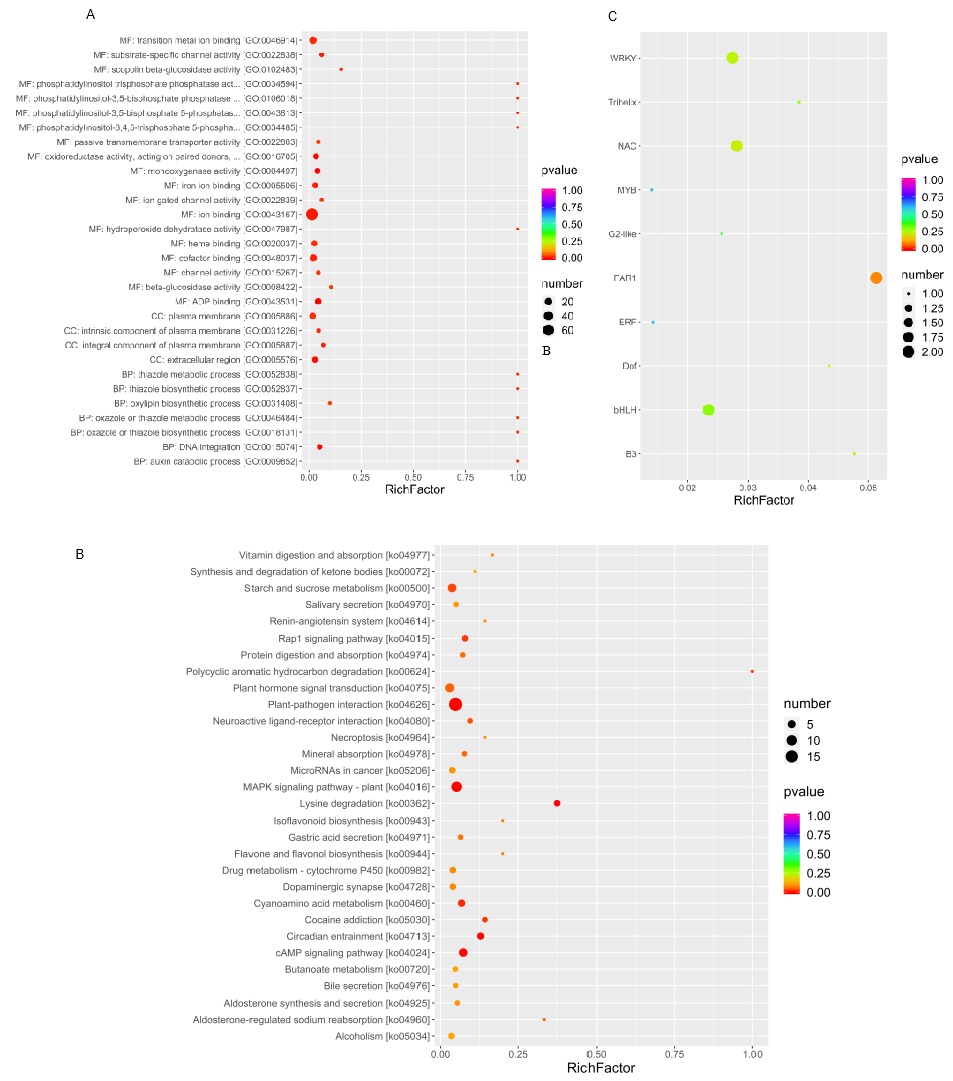

Supplement: Supplementary file 2 — Additional file 2 : Figure S2. Bioinformatic analysis of differentially expressed genes in NPB-Piz-t compared to NPB in response to M. oryzae KJ201. A, GO analysis of differentially expressed genes of NPB-Piz-t compared to NPB. B, KEGG analysis of differentially expressed genes of NPB-Piz-t compared to NPB. C, Transcript factor of differentially expressed genes. The results are summarized in three main categories: biological process, cellular component, and molecular function. [file 12284_2020_439_MOESM2_ESM.tif]

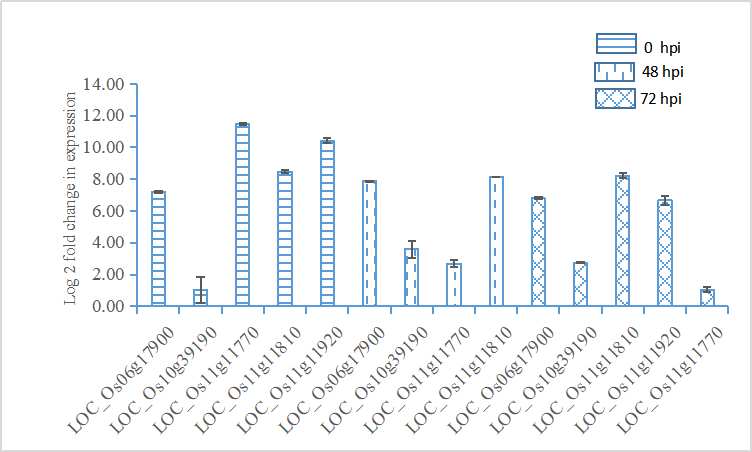

Supplement: Supplementary file 3 — Additional file 3 : Figure S3. The qRT-PCR validation of the differentially expressed gene of between NPB-Piz-t and NPB inoculated with KJ201 at 0, 48 and 72 hpi in the pink module. The data were log2 transformed for FC. [file 12284_2020_439_MOESM3_ESM.tif]
