# Supplementary material for: Weighted Gene Co-Expression Network Coupled with a Critical-Time-Point Analysis during Pathogenesis for Predicting the Molecular Mechanism Underlying Blast Resistance in Rice
Source: Rice (N Y). 2020 Dec 11;13:81. doi: 10.1186/s12284-020-00439-8 (PMC7732884; doi:10.1186/s12284-020-00439-8)
Supplement: Supplementary file 5 — Additional file 5 : Table S1. Primers used for real-time quantitative PCR used in the study. [file 12284_2020_439_MOESM5_ESM.doc]

**Table S1.** Primers used for real-time quantitative PCR

| **Gene ID.** | **Forward primer (5' - 3')** | **Reverse primer(5' - 3')** |
| --- | --- | --- |
| LOC_Os02g41710 | ATGTGCATCGAGCTGAAGTA | AGCTTCGGTATCACAATCCA |
| LOC_Os02g47990 | TTGGCTCTGGTATGAGTGGA | GAGGTTTGGGGCTAGGTATT |
| LOC_Os04g27096 | TGGATAGATTCCGAGACGCG | TATTGGGGAGCAAGAAGAGCA |
| LOC_Os06g17900 | TGCAGGTTGGAATCTGGGA | TCGTCGTCAACGTGATCACC |
| LOC_Os06g17920 | ACTAAGACGGGATTCAGGGCT | GTGGCAGTGGATCTGGCAA |
| LOC_Os07g03279 | AGTAGCACTTGCGGCAGCCA | GAGCCTCCGAAGAGGTTCTCG |
| LOC_Os07g03319 | GGTCGGCTTGTGGATGGAGG | CGGGCGGGAAATAGTTGCAG |
| LOC_Os07g03368 | GGCTACGGCGAGAACCTCTT | TCGTGCGGCTCCACATCA |
| LOC_Os07g03499 | GGCGGACTACGTGTACAGCA | CGGGCGGGAAATAGTTGCAG |
| LOC_Os07g03600 | CCACTACACGCAGGTGGTGTGG | AGTAAGGGCGCTGATCGGGC |
| LOC_Os10g39190 | TCGCACTCGCACCACCATCA | GGCACCGACTTGAGCACCACA |
| LOC_Os11g02379 | CATGGTGGATGGAGGAGGA | GACACCTATGAGCTGAGCCA |
| LOC_Os11g02520 | GCCATTACTTCCGAGCGACA | AGGTGCTTGAGCCACAGTTG |
| LOC_Os11g11770 | GCAAAGTCGTTTCCGCTG | ATGCCTTCAGGGACGCTCT |
| LOC_Os11g11810 | TAACTTACGGTACATTGGCCT | CACTCCTTCGAAATACCGGAA |
| LOC_Os11g11920 | AGAAGCCGTCGGTGTTTGAC | AGCAGGAGCATTTCGTGGAG |
| LOC_Os11g11990 | AATGATAGTGTTGCTGCCAC | CAAGCCAATGTAACGCAGA |
| LOC_Os11g43700 | AGGCAGCGGTATATTAGCAT | ACTGTAACTTTGCGTGTCGT |
| LOC_Os12g17430 | CATCCATGGCCCTATGGAGA | TGTGCCCACCTTCTACTTGTT |
| Ubiquitin | AACCAGCTGAGGCCCAAGA | ACGATTGATTTAACCAGTCCATGA |
