# Supplementary material for: Weighted Gene Co-Expression Network Coupled with a Critical-Time-Point Analysis during Pathogenesis for Predicting the Molecular Mechanism Underlying Blast Resistance in Rice
Source: Rice (N Y). 2020 Dec 11;13:81. doi: 10.1186/s12284-020-00439-8 (PMC7732884; doi:10.1186/s12284-020-00439-8)
Supplement: Supplementary file 3 [file 12284_2020_439_MOESM4_ESM.doc]

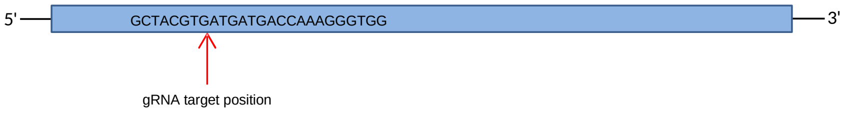

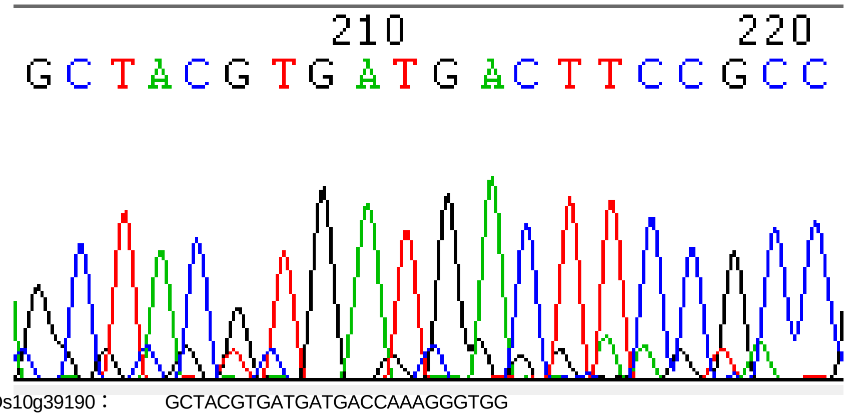

LOC_Os10g39190：　GCTACGTGATGATGACCAAAGGGTGG

LOC_Os10g39190-KO#1 : GCTACGTGATGATGAC---

---TTCCGCC

Reference:　GCTACGTGATGATGACCAAAGGGTGGAGCCGCTTCGTCAAGGAGAAGCGCCTCGGCGCCGGCGACA CCGTGTCGTTCGGCCGCGGCCTCGGCGACGCCGCCCGCGGCCGCCTCTTCATCGACTTCCGCC

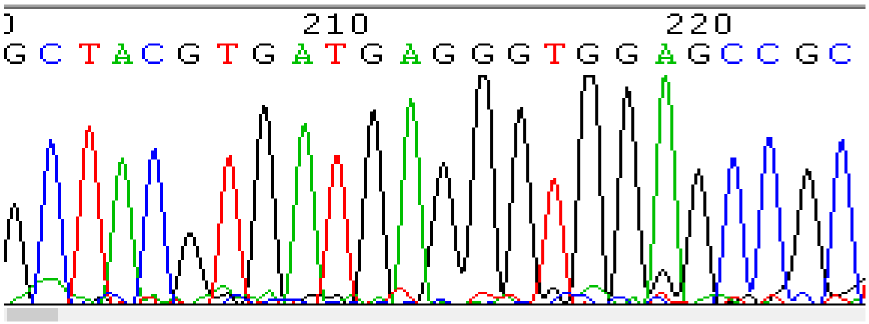

LOC_Os10g39190：　GCTACGTGATGATGACCAAAGGGTGG

LOC_Os10g39190-KO#2 : GCTACGTGATGA-----GGGTGG

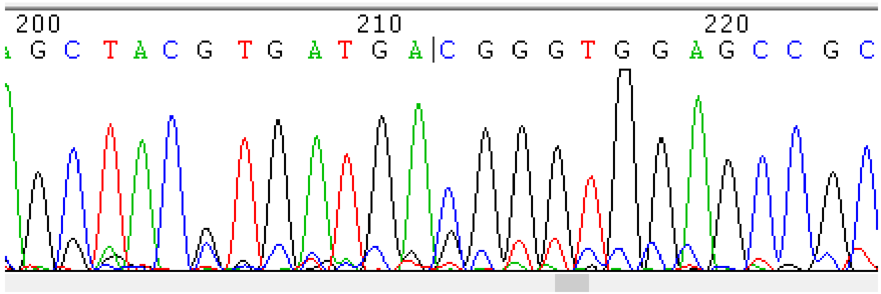

LOC_Os10g39190：　GCTACGTGATGATGACCAAAGGGTGG

LOC_Os10g39190-KO#3 : GCTACGTGATGATGAC----GGGTGG
